# Supplementary material for: Architecture of a SARS-CoV-2 mini replication and transcription complex
Source: Nat Commun. 2020 Nov 18;11:5874. doi: 10.1038/s41467-020-19770-1 (PMC7675986; doi:10.1038/s41467-020-19770-1)
Supplement: Supplementary file 1 — Supplementary Information [file 41467_2020_19770_MOESM1_ESM.pdf]

Supplementary Information for:

Architecture of a SARS-CoV-2 mini replication and transcription complex

Yan et al.

Contents **Page**

Supplementary Figures **2**

Supplementary Figure 1 2

Supplementary Figure 2 3

Supplementary Figure 3 5

Supplementary Figure 4 6

Supplementary Figure 5 7

Supplementary Figure 6 8

Supplementary Figure 7 9

Supplementary Figure 8 10

Supplementary Tables **11**

Supplementary Table 1. Cryo-EM data statistics 11

Supplementary Table 2. Interactions of nsp13 with nsp7-nsp8-nsp12 complex. 12

Supplementary Table 3. Interactions of nsp13-1 and nsp13-2. 12

Supplementary Table 4. Comparison of H/D exchange mass spectrometry (HDX MS) of nsp13-RNA[a] and structural interactions of nsp13-template RNA 13

Supplementary Table 5. List of primers used for SARS-CoV-2 protein expression 13

Supplementary References **14**

Supplementary Figures

Supplementary Figure 1

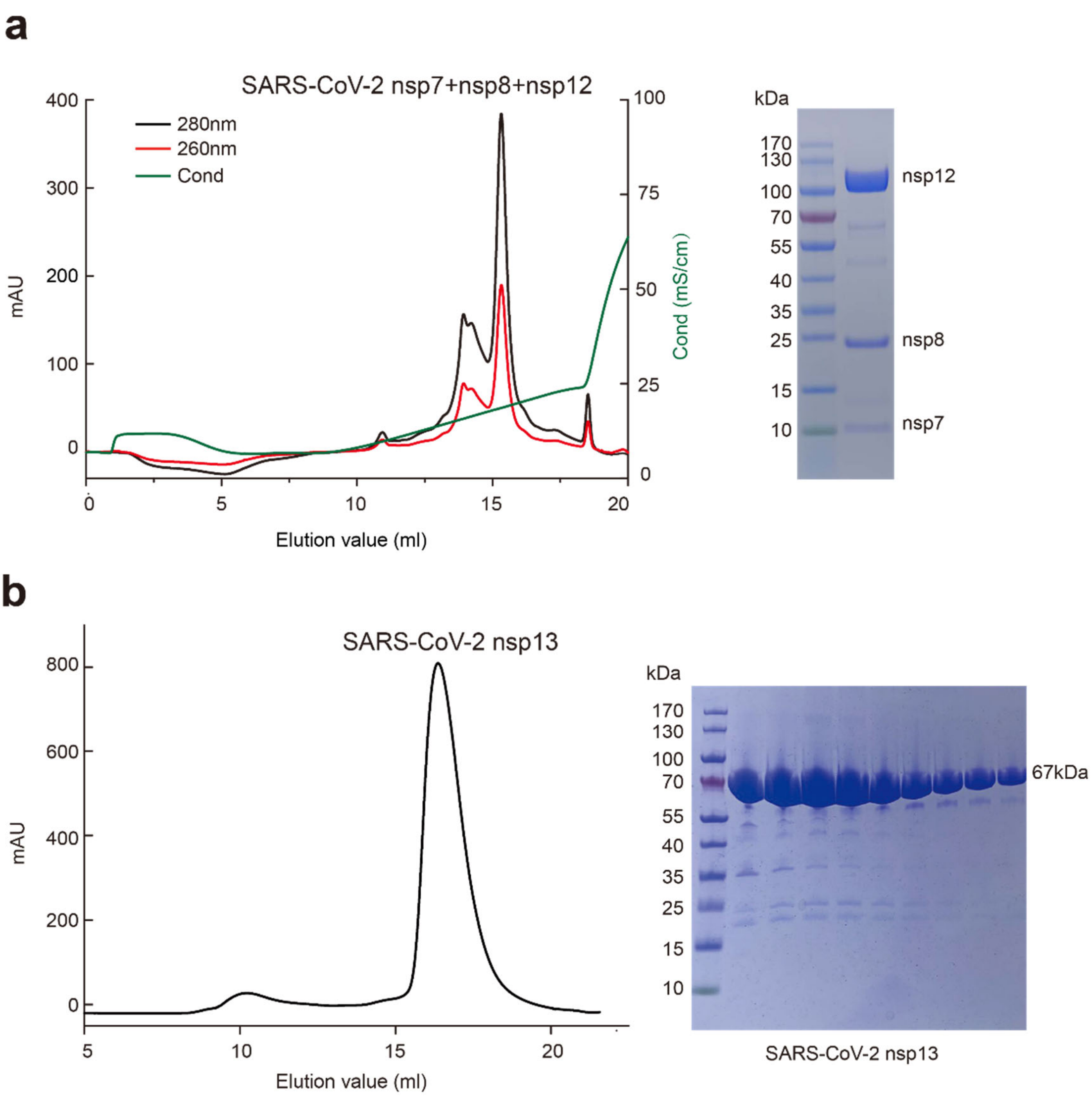

Supplementary Figure 1. The purification of SARS-CoV-2 nsp12-nsp7-nsp8 complex and nsp13. Anion exchange chromatography of the nsp12-nsp7-nsp8 complex **(a)** and size-exclusion chromatogram of the affinity-purified SARS-CoV-2 nsp13 **(b)**. Data from a mono-Q 5/50 **(a)** and Superdex 200 10/30 column **(b)** are shown in black. The target proteins were analyzed by the SDS-PAGE. The standard protein markers are shown lane with labels. The lane labels in the SDS-PAGE with blue color indicate the peak positions. All proteins and nsp12-nsp7-nsp8 complex can be stablely expressed and purified and the data presented are repeated at least three times with similar results.

Supplementary Figure 2

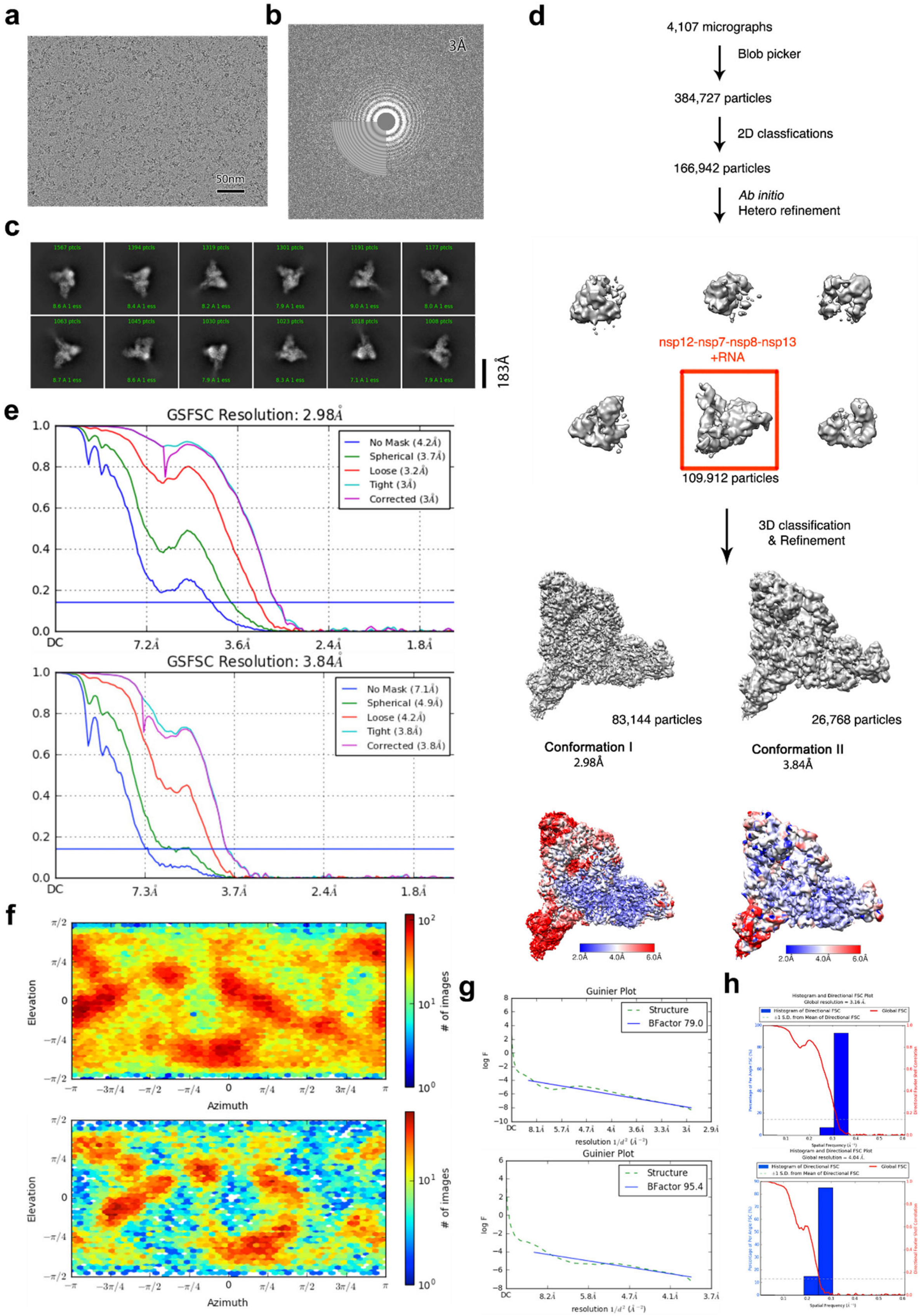

Supplementary Figure 2. Cryo-EM reconstruction. (a) Raw image of SARS-CoV-2 nsp7-8_2-12-13_2 complex particles in vitreous ice recorded at defocus values of -2.0 to -1.0 μm. Scale bar is 50 nm. **(b)** Power spectrum of the image shown in (a), with plot of the rotationally averaged intensity versus resolution. White circle indicates the spatial frequency corresponding to 3.0 Å resolution. **(c)** Representative class averages. The edge of each square is 386 Å, approximately. **(d)** The data processing scheme used to obtain the final map and the overview of SARS-CoV-2 nsp7-8_2-12-13_2 complex density maps in 2 conformations. Local resolutions are colored in blue-grey-red as shown in color key. **(e)** Golden-Standard fourier shell correlations (GSFSC) of the final 3D reconstruction following gold standard refinement. FSC curves are plotted before (red) and after (green) masking in addition to post-correction (blue), accounting for the effect of the mask using phase randomization. **(f)** The viewing direction distributions. **(g)** Map-sharpening B-factor is estimated in Guinier plots. **(h)** The 3DFSC sphericity was analyzed with threeDFSC in cryoSPARC. Subfigure **e**, **f**, **g**, **h** are plotted from dataset of conformation I and II, respectively. The data presented are repeated at least three times with similar results.

Supplementary Figure 3

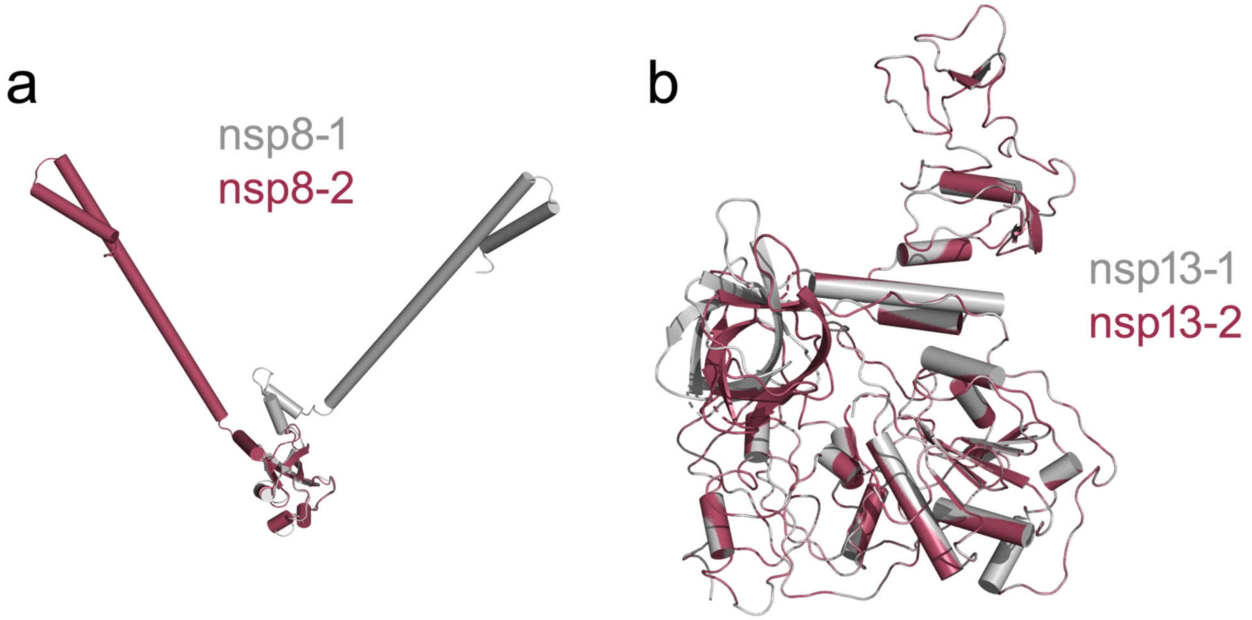

Supplementary Figure 3. Comparison of nsp8 and nsp13 molecules in the mini RTC. The structures of two nsp8 molecules **(a)** and two nsp13 molecules are aligned **(b)** and shown in the same orientation. Structures of nsp8 and nsp13 are shown by using they are in form 1 mini RTC as representative.

Supplementary Figure 4

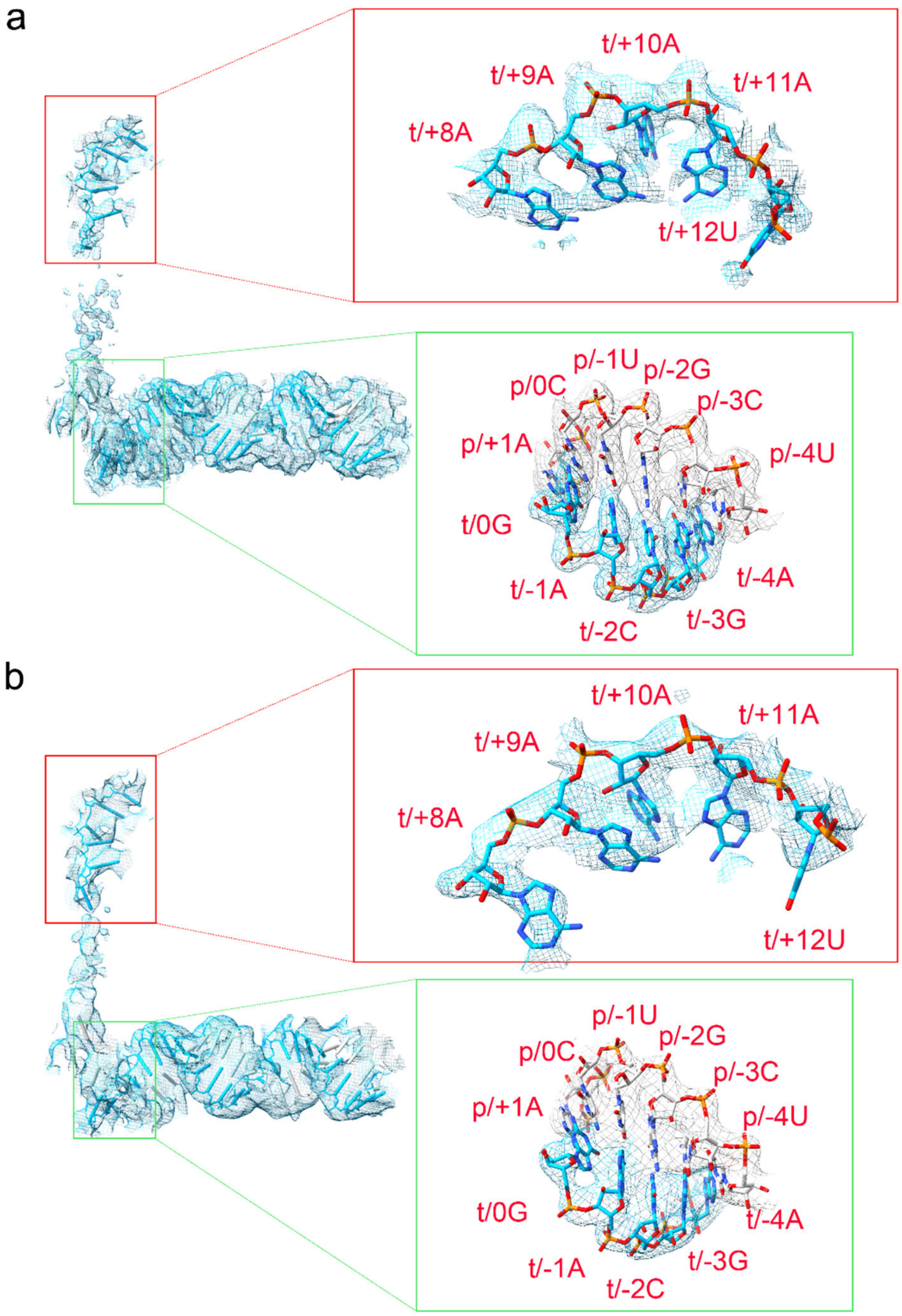

Supplementary Figure 4. Densities RNA bound in mini RTC. RNA bound to form 1 **(a)** and form 2 **(b)** mini RTCs are shown as colored stick with the color scheme in Figure 1. The cryo-EM densities covering RNA are displayed as grey meshes. Enlarged parts are presented in the right panels. The sigma levels used in **(a)**: left panel, 4.95 rmsd; upper right

panel, 4.31 rmsd; bottom right panel, 8.61 rmsd. The sigma levels used in **(b)**: left panel, 5.41 rmsd; upper right panel, 8.11 rmsd; bottom right panel, 13.52 rmsd.

Supplementary Figure 5

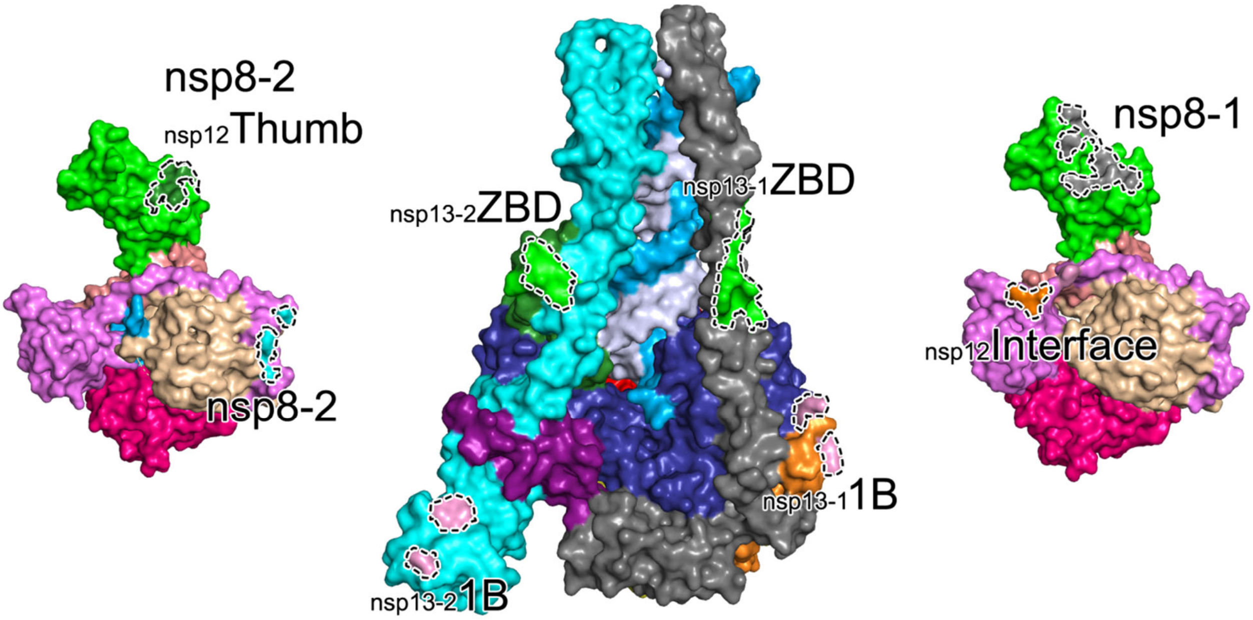

Supplementary Figure 5. Interaction between two nsp13 molecules and nsp7-nsp8-nsp12. All proteins are covered by molecular surface with the same color scheme in Figure 1. The interfaces on each protein are framed out with the colors of their interacting partners. The labels indicate the interacting partners of the corresponding regions.

Supplementary Figure 6

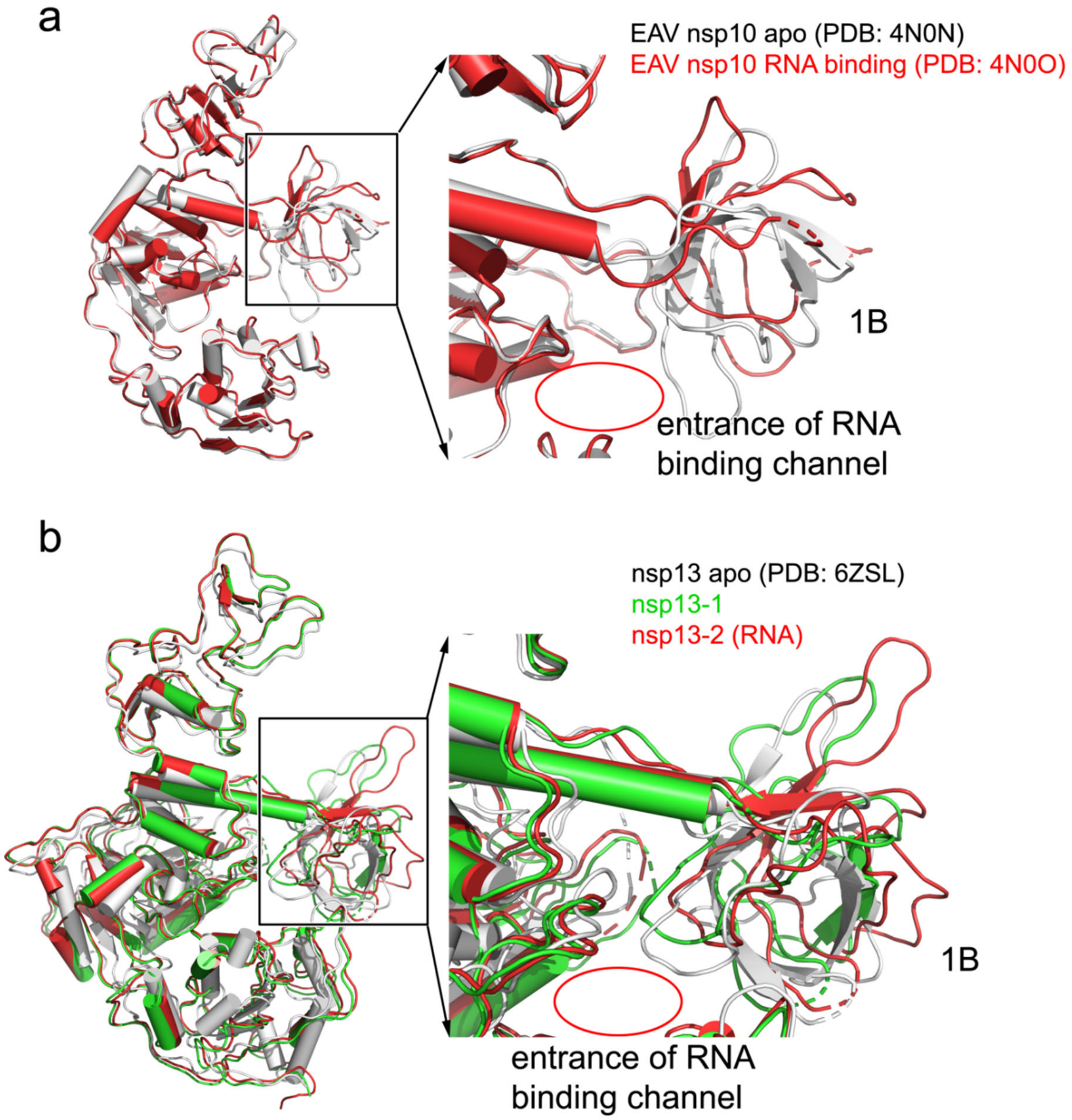

Supplementary Figure 6. Comparison of nidoviral helicases. (a) Structures of EAV helicase (nsp10) in apo form (PDB: 4N0N) and in RNA binding form (PDB: 4N0O)[1], and **(b)** structures of SARS-CoV-2 nsp13 in apo form (PDB: 6ZSL), nsp13-1 and nsp13-2 in the mini RTC, are aligned and shown in the same orientation. Framed parts are enlarged in the right panels.

Supplementary Figure 7

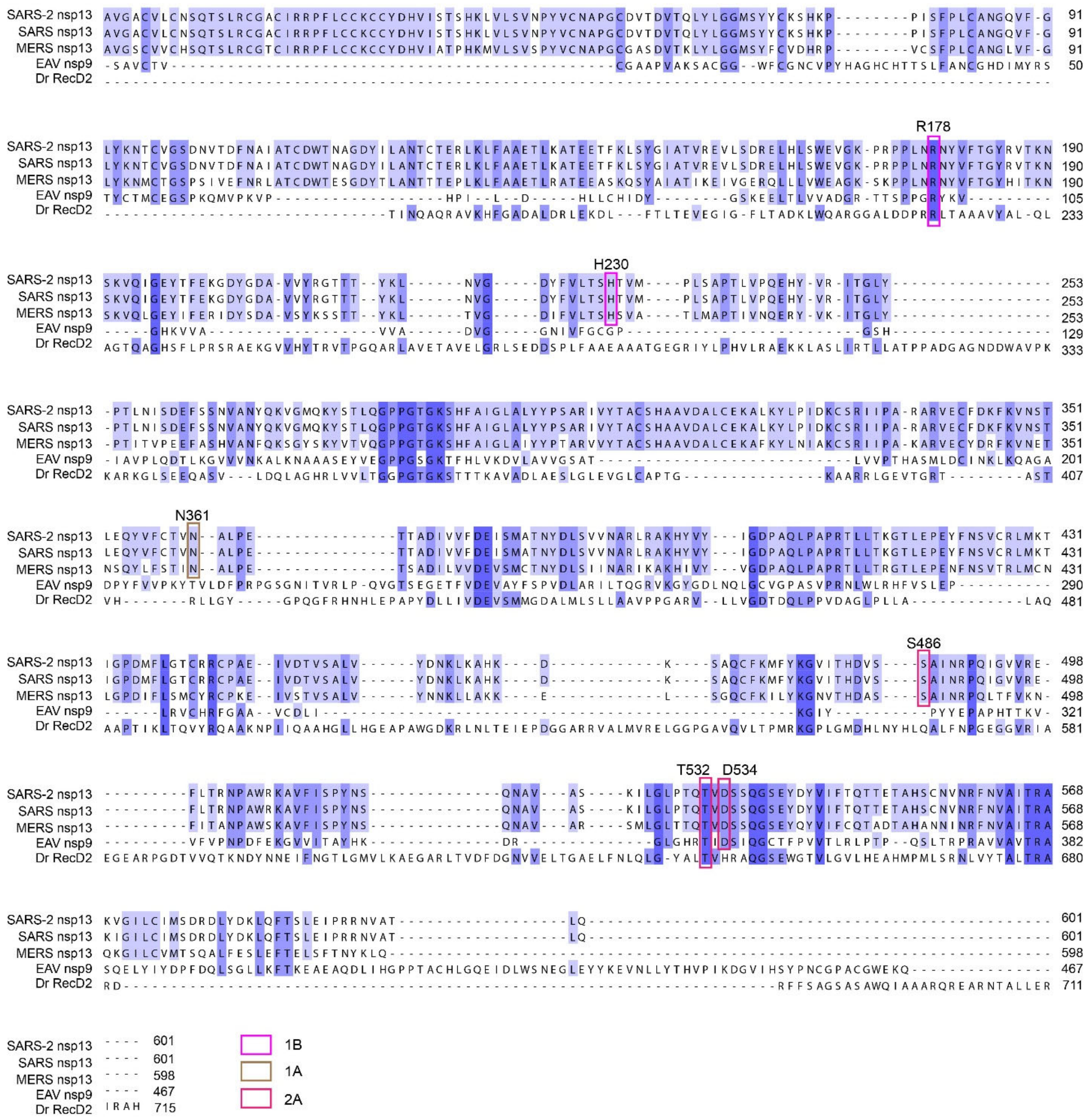

Supplementary Figure 7. Sequence alignment of the helicases in different species, helicase nsp13 encoded by SARS-CoV-2, SARS-CoV, MERS-CoV, nsp9 helicase from EAV and RecD2 of Deinococcus radiodurans. The residues with blue, light blue or white backgrounds indicate the identical, conserved or non-conserved residues. Nsp13 conservative residues are also indicated with the same color scheme as that in Figure 1b.

Supplementary Figure 8

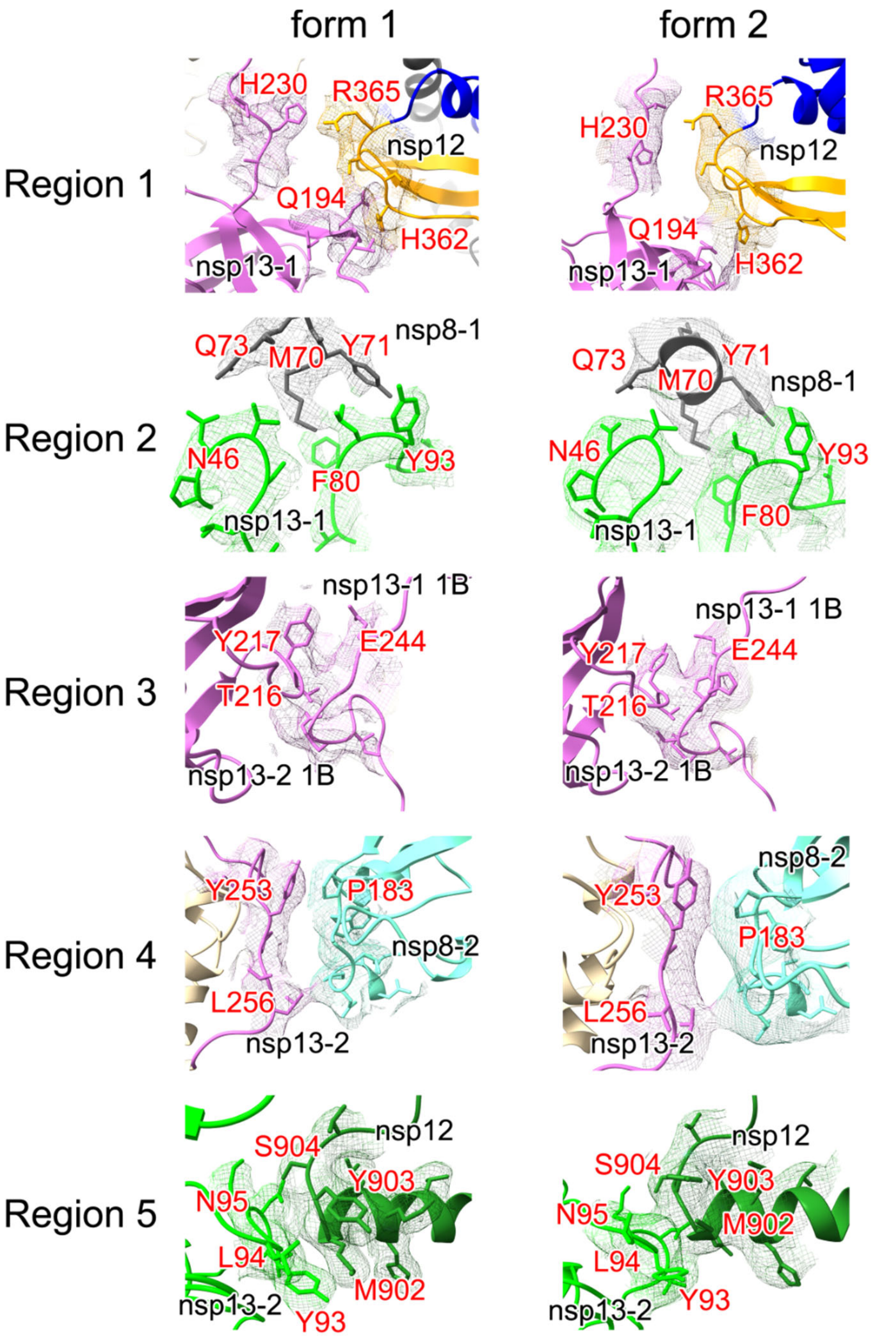

Supplementary Figure 8. Densities for the interaction regions (related to Figure 3). Interaction regions 1-5 in form 1 and form 2 mini RTC are covered by cryo-EM densities. The color scheme is same as in Figure 3. The cryo-EM densities are shown as colored meshes.

Supplementary Tables

Supplementary Table 1. Cryo-EM data statistics

	Form 1	From 2
**Data collection and processing**		
Magnification	290,000x	290,000x
Voltage (kV)	300	300
Electron exposure (e–/Å^2)	60	60
Defocus range (μm)	-2.0 to -1.0	-2.0 to -1.0
Pixel size (Å)	0.82	0.82
Symmetry imposed	C1	C1
Initial particle images (no.)	384,727	384,727
Final particle images (no.)	83,144	26,768
Map resolution (Å)	2.98	3.84
FSC threshold	0.143	0.143
Map resolution range (Å)	1.8 to 11.5	2.0 to 9.0
**Refinement**		
Model resolution range (Å)	∞ to 2.9	∞ to 3.8
Map sharpening *B* factor (Å^2)	-79.0	-95.4
Model composition		
Non-hydrogen atoms	21,086	21,086
Protein residues	2,542	2,542
Ligands	57	56
B factors (Å^2)		
Protein	58.0	72.0
Ligand	84.0	92.0
R.m.s. deviations		
Bond lengths (Å)	0.004	0.008
Bond angles (°)	0.600	0.670
Validation		
MolProbity score	2.42	2.52
Clashscore	14	18
Poor rotamers (%)	0.00	0.00
Ramachandran plot		
Favored (%)	92.04	90.1
Allowed (%)	7.76	9.4
Disallowed (%)	0.2	0.5

Supplementary Table 2. Interactions of nsp13 with nsp7-nsp8-nsp12 complex.

Molecule	Residue	Molecule	Residue
nsp13-1	V45	nsp8-1	Q73
	N46		Q73
	M68		Q69
	I79		M55, K58, L59
	S80		L59
	F81		L59, A63
	F90		M67
	G91		M67, M70
	L92		M70, Y71
	Y93		Y71
	V193	nsp12	H362
	Q194		H362, S363
	H230		R365
nsp13-2	R248	nsp8-2	D134
	Y253		P133
	L256		P178
	L92	nsp12	M902, Y903
	Y93		D901, M902
	K94		S904
	N95		S904

Supplementary Table 3. Interactions of nsp13-1 and nsp13-2.

Molecule	Residue	Molecule	Residue
nsp13-1	E244	nsp13-2	Y217
	H245		Y217, K218
	Y246		T216, K218
	V247		D160, Y211, T216, Y217, K218
	R248		T216
	T250		T216

Supplementary Table 4. Comparison of H/D exchange mass spectrometry (HDX MS) of nsp13-RNA[a] and structural interactions of nsp13-template RNA .

Nsp13 domain	HDX MS peptides	nsp13-2 and RNA
1B	T153-N179, D209-G224	R178，H230
1A	A318-D330, S331-F357, C358-D369	N361
2A	V496-F511, S523-D542, T547-C574	S486, T532, D534

[a] HDX MS of nsp13 and RNA has been published by our group in 2019[2].

Supplementary Table 5. List of primers used for SARS-CoV-2 protein expression.

Primer		Sequence 5' - 3'
1	nsp12-Nde-F	GGAATTCCATATGGGTTCCGCTGACGCCCAG
2	nsp12-Xho-R	CTCGAGCGGTTAATGATGATGATGATGATGATGATGATGAT GCTGCAGCACGGTGTGTGGAG
3	nsp12-R365A-BamHI-F	AGGACGTGAACCTGCACTCCAGCGCATTGTCATTCAAG
4	nsp12-R365A-XhoI-R	AGACCAGCAGTTCCTTGAATGACAATGCGCTGGAGTGCAG
5	nsp13-BamH-F	CGCGGATCCATGGCTGTTGGGGCTTGTGTTCTTTGC
6	nsp13-XhoI-R	CCGCTCGAGTTATTGTAAAGTTGCCACATTCCTACG
7	nsp13-T216A-BamH-F	GTTGTGTACAGAGGTACTACGGCATACAAGTTGAAT
8	nsp13-T216A-XhoI-R	TCACCAACATTCAACTTGTATGCCGTAGTACCTCT
9	nsp8-BamHI-F	CGCGGATCCGCTATAGCCTCAGAGTTTAG
10	nsp8-XhoI-R	CCGCTCGAGTTACTGTAATTTGACAGCAGAATTGG
11	nsp7-NdeI-F	GGAATTCCATATGTCTAAAATGTCAGATGTAAAGTGC
12	nsp7-XhoI-R	CTCGAGCGGTTATTGTAAGGTTGCCCTGTTGTCC

.

Supplementary References

1 Deng, Z. *et al.* Structural basis for the regulatory function of a complex zinc-binding domain in a replicative arterivirus helicase resembling a nonsense-mediated mRNA decay helicase. *Nucleic acids research* **42**, 3464-3477, doi:10.1093/nar/gkt1310 (2014).

2 Jia, Z. *et al.* Delicate structural coordination of the Severe Acute Respiratory Syndrome coronavirus Nsp13 upon ATP hydrolysis. *Nucleic acids research* **47**, 6538-6550, doi:10.1093/nar/gkz409 (2019).
