# Supplementary material for: Architecture of a SARS-CoV-2 mini replication and transcription complex
Source: Nat Commun. 2020 Nov 18;11:5874. doi: 10.1038/s41467-020-19770-1 (PMC7675986; doi:10.1038/s41467-020-19770-1)
Supplement: Supplementary file 3 — Reporting Summary [file 41467_2020_19770_MOESM3_ESM.pdf]

nature research

Corresponding author(s): Zhiyong Lou

Last updated by author(s): Oct 27, 2020

Reporting Summary

Nature Research wishes to improve the reproducibility of the work that we publish. This form provides structure for consistency and transparency in reporting. For further information on Nature Research policies, see our Editorial Policies and the Editorial Policy Checklist.

Statistics

For all statistical analyses, confirm that the following items are present in the figure legend, table legend, main text, or Methods section.

n/a	Confirmed	
☐	☒	The exact sample size (*n*) for each experimental group/condition, given as a discrete number and unit of measurement
☐	☒	A statement on whether measurements were taken from distinct samples or whether the same sample was measured repeatedly
☒	☐	The statistical test(s) used AND whether they are one- or two-sided<br>*Only common tests should be described solely by name; describe more complex techniques in the Methods section.*
☒	☐	A description of all covariates tested
☒	☐	A description of any assumptions or corrections, such as tests of normality and adjustment for multiple comparisons
☒	☐	A full description of the statistical parameters including central tendency (e.g. means) or other basic estimates (e.g. regression coefficient) AND variation (e.g. standard deviation) or associated estimates of uncertainty (e.g. confidence intervals)
☒	☐	For null hypothesis testing, the test statistic (e.g. *F*, *t*, *r*) with confidence intervals, effect sizes, degrees of freedom and *P* value noted<br>*Give P values as exact values whenever suitable.*
☒	☐	For Bayesian analysis, information on the choice of priors and Markov chain Monte Carlo settings
☒	☐	For hierarchical and complex designs, identification of the appropriate level for tests and full reporting of outcomes
☒	☐	Estimates of effect sizes (e.g. Cohen's *d*, Pearson's *r*), indicating how they were calculated

Our web collection on statistics for biologists contains articles on many of the points above.

Software and code

Policy information about availability of computer code

Data collection	1. Serial EM 2. Perkin-Elmer Enspire
Data analysis	1.BiacoreT200 2.MotionCor2 3.RELION 3.0 4.CTFFIND4 5.THUNDER 6.UCSF Chimera 7.Coot 8.Phenix 9.GraphPad Prism 10.cryoSPARC

For manuscripts utilizing custom algorithms or software that are central to the research but not yet described in published literature, software must be made available to editors and reviewers. We strongly encourage code deposition in a community repository (e.g. GitHub). See the Nature Research guidelines for submitting code & software for further information.

Data

Policy information about availability of data

All manuscripts must include a data availability statement. This statement should provide the following information, where applicable:

- Accession codes, unique identifiers, or web links for publicly available datasets
- A list of figures that have associated raw data
- A description of any restrictions on data availability

web links for publicly available datasets: PDB, NCBI, pubmed Accession codes: The cryo-EM density maps and the structures were deposited into the Electron Microscopy Data Bank (EMDB) and Protein Data Bank (PDB) with the following accession numbers: form 1, EMD-30492 and 7CXM; form 2, EMD-30493 and 7CXN; Figures with raw data: 1. Figure 1c has raw data.

Field-specific reporting

Please select the one below that is the best fit for your research. If you are not sure, read the appropriate sections before making your selection.

☒ Life sciences ☐ Behavioural & social sciences ☐ Ecological, evolutionary & environmental sciences

For a reference copy of the document with all sections, see nature.com/documents/nr-reporting-summary-flat.pdf

Life sciences study design

All studies must disclose on these points even when the disclosure is negative.

Sample size	1.predict the molecular weight of sample by calculating using ExPASy<br>2.run SDS-PAGE to confirm the size of sample compared to protein marker<br>3.measure the diameter of sample using negative staining
Data exclusions	None
Replication	use RELION 3.0 and THUNDER to refine the electronic density map of the final structure repeatedly to make sure the accuracy and reproductivity of the result
Randomization	1.The protein purification, cell culture, the preparation of cryo-EM sample and data collection were performed following the standard procedure.<br>2.use random point to pick particles from images to avoid artificial tendency
Blinding	The investigators were acknowledged the samples when they collected cryo-EM data and performed validation. Because we didn't select and exclude data manually for further analyses, this is not relevant with our study and conclusion.

Reporting for specific materials, systems and methods

We require information from authors about some types of materials, experimental systems and methods used in many studies. Here, indicate whether each material, system or method listed is relevant to your study. If you are not sure if a list item applies to your research, read the appropriate section before selecting a response.

Materials & experimental systems

n/a	Involved in the study
☒	☐ Antibodies
☒	☐ Eukaryotic cell lines
☒	☐ Palaeontology and archaeology
☒	☐ Animals and other organisms
☒	☐ Human research participants
☒	☐ Clinical data
☒	☐ Dual use research of concern

Methods

n/a	Involved in the study
☒	☐ ChIP-seq
☒	☐ Flow cytometry
☒	☐ MRI-based neuroimaging
